# Exploring Congolese refugees’ experiences with abortion care in Uganda: a multi-methods qualitative study

**DOI:** 10.1080/26410397.2019.1681091

**Published:** 2019-11-18

**Authors:** Ruth Nara, Amanda Banura, Angel M. Foster

**Affiliations:** a Former student, Faculty of Health Sciences, University of Ottawa, Ottawa, Canada; b Student, Faculty of Science, Uganda Martyrs University, Kampala, Uganda; c Professor, Faculty of Health Sciences, University of Ottawa, Ottawa, Canada; d Principal Scientist, Institute of Population Health, University of Ottawa, Ottawa, Canada

**Keywords:** abortion, Africa, the Democratic Republic of the Congo, post-abortion care, refugees, Uganda

## Abstract

Uganda hosts 1.4 million refugees and conflict-affected people. Widely regarded as the best place in Africa to be a refugee, Uganda’s policies encourage self-sufficiency and local integration. However, abortion is legally restricted and recent studies suggest that displaced women and girls have persistent unmet sexual and reproductive health needs. In 2017, we conducted a multi-methods study to assess the reproductive health needs of displaced Congolese women in camp- and urban-based settings in Uganda. Our project focused on maternal health and delivery care, contraception, and abortion/post-abortion services and the intersection of these issues with sexual and gender-based violence. We interviewed 11 key informants, facilitated 4 focus group discussions with refugee women, and conducted 21 in-depth interviews with Congolese women of reproductive age to understand better knowledge, attitudes, practices, and services. Using both inductive and deductive techniques, we employed a multi-phased analytic plan to identify content and themes and triangulate and interpret findings. Our results suggest that Congolese refugees in Uganda are unable to navigate the legal restrictions on abortion and are engaging in unsafe abortion practices. This appears to be the case for those living in both camps and urban areas. The legal restrictions on induced abortion pose a barrier to the provision of post-abortion care. Efforts to ensure access to comprehensive abortion care should be prioritised and providing information and support to women in need of post-abortion care is imperative.

## Résumé

L’Ouganda abrite 1,4 million de réfugiés et de personnes touchées par un conflit. Largement considéré comme le meilleur endroit en Afrique pour les réfugiés, l’Ouganda applique des politiques qui encouragent l’autosuffisance et l’intégration sur place. Néanmoins, l’avortement est limité par la loi et de récentes études suggèrent que les besoins en santé sexuelle et reproductive des femmes et filles déplacées demeurent non satisfaits. En 2017, nous avons mené une étude à méthodes multiples pour évaluer les besoins de santé reproductive des Congolaises déplacées dans des camps et en milieu urbain en Ouganda. Notre projet se centrait sur la santé maternelle et les soins obstétricaux, la contraception et les services d’avortement/après avortement ainsi que sur l’intersection de ces questions avec la violence sexuelle et sexiste. Nous avons interrogé 11 informateurs clés, animé quatre discussions par groupe d’intérêt avec des réfugiées et réalisé 21 entretiens approfondis avec des Congolaises en âge de procréer pour mieux comprendre les connaissances, les attitudes, les pratiques et les services. À l’aide de techniques inductives et déductives, nous avons employé un plan analytique à phases multiples pour identifier le contenu et les thèmes, puis trianguler et interpréter les résultats. Nos conclusions donnent à penser que les réfugiées congolaises en Ouganda sont incapables de se frayer un chemin à travers les restrictions juridiques sur l’avortement et se livrent à des pratiques d’avortement à risque. Cela semble être le cas pour celles qui vivent dans les camps et en milieu urbain. Les restrictions juridiques sur l’interruption de grossesse posent un obstacle à la prestation de soins après l’avortement. Des activités pour garantir l’accès à des soins d’avortement complets devraient être prioritaires et il est impératif de fournir des informations et de soutenir les femmes qui ont besoin de soins après un avortement.

## Resumen

Uganda acoge a 1.4 millones de refugiados y personas afectadas por conflicto. Reconocido ampliamente como el mejor lugar en África para ser refugiado, Uganda tiene políticas que fomentan la autosuficiencia y la integración local. Sin embargo, el aborto es restringido por la ley y recientes estudios indican que las mujeres y niñas desplazadas tienen persistentes necesidades insatisfechas de salud sexual y reproductiva. En 2017, realizamos un estudio multimétodo para evaluar las necesidades de salud reproductiva de mujeres congolesas desplazadas en campos de refugiados y zonas urbanas de Uganda. Nuestro proyecto se enfocó en servicios de salud materna y atención durante el parto, anticoncepción y aborto/atención postaborto, y en la intersección de estos asuntos con la violencia sexual y la violencia de género. Entrevistamos a 11 informantes clave, facilitamos 4 discusiones en grupos focales con mujeres refugiadas y realizamos 21 entrevistas a profundidad con mujeres congolesas en edad reproductiva para entender mejor los conocimientos, actitudes, prácticas y servicios. Utilizando técnicas inductivas y deductivas, empleamos un plan analítico multifacético para identificar contenido y temáticas, y triangular e interpretar los hallazgos. Nuestros resultados indican que las refugiadas congolesas en Uganda no pueden navegar las restricciones legislativas impuestas al aborto y están participando en prácticas de aborto inseguro. Ese parece ser el caso de las mujeres que viven en campos o en zonas urbanas. Las restricciones legislativas impuestas al aborto inducido representan una barrera para la prestación de servicios de atención postaborto. Se debe dar prioridad a los esfuerzos por garantizar acceso a los servicios de atención integral del aborto y es imperativo proporcionar información y apoyo a las mujeres que necesitan atención postaborto.

## Introduction

By the end of 2018, the global humanitarian crisis left 70.8 million people displaced, the majority of whom are hosted in low-income and lower-middle-income countries.^1^ Trends suggest that the number of those in need of humanitarian assistance, including health, food, security, and protection services, continues to grow.^2^ Women and girls constitute a large portion of this population and are often vulnerable to sexual violence and exploitation.^3^ Moreover, the systemic use of sex as a weapon of war during conflicts increases the risk of unintended pregnancy.

Despite the overall reduction in global maternal mortality and the increase in the provision of sexual reproductive health (SRH) services to displaced populations,^3–4^ unintended pregnancy is a continued problem.^5^ Recent estimates suggest that 44% of pregnancies in 2010–2014 were unintended worldwide.^6^ More than 50% of these unintended pregnancies ended in abortion.^6^ In areas where abortion is legally restricted, women often turn to unsafe methods of pregnancy termination^7^ and are at considerable risk of negative reproductive health outcomes, including disability and death.^5,8^

Reliable information about abortion in refugee and conflict-affected settings is limited. However, displaced populations are recognised as being at heightened risk of sexual violence, lacking access to ongoing contraceptive methods and experiencing changes in pregnancy intentions, dynamics that increase the risk of unintended pregnancy.^9^ Consequently, refugees and displaced women who reside in countries where abortion is legally restricted face considerable barriers accessing safe abortion care.^10^ Evidence indicates that refugee and displaced women are employing unsafe practices to terminate unwanted pregnancies.^11–14^ Even when they are eligible for safe and legal abortion care, refugee women may not be able to navigate local health systems to obtain services.^15–17^

Refugees and displaced populations living in Uganda have limited access to abortion care. Article 22(2) of the Ugandan Constitution states: “No person has the right to terminate the life of an unborn child except as may be authorized by law”.^18^ However, this law has not been enforced since 1995 and, consequently, there is significant reliance on the provisions of the Penal Code Act 1950.^19–22^ According to the Penal Code, attempting to abort a pregnancy is subject to 14 years in prison, attempting to induce a miscarriage is subject to seven years in prison, and providing a medication that induces an abortion is subject to three years of prison.^23^ However, the National Policy Guideline and Service Standard for Sexual and Reproductive Health and Rights issued in 2006 allows for exceptions under which abortion can be provided. This policy stipulates that termination of a pregnancy is permissible in cases in which the pregnancy threatens the life of the woman, involves a fetal anomaly, was the result of rape or incest, or is that of an HIV positive woman.^19,21^ The Ugandan Ministry of Health indicates that these exceptions are subject to interpretation. The inconsistencies and ambiguities in the various laws and policy mean that women and providers alike are often unaware of the circumstances when an abortion is legally permitted.^21^ These policies impact both Ugandan and displaced populations living in the country and the prevalence of unsafe abortion is high.^24^

Uganda is increasingly welcoming refugees and has become the largest refugee-hosting country in Africa. The country is considered to be one of the most hospitable places to seek asylum in the region as it has progressive policies, including offering refugees free education and healthcare.^25–26^ Of the nearly 1.4 million refugees from 13 different countries living in Uganda,^27–28^ approximately 317,000 are from the Democratic Republic of the Congo (DRC). Many displaced Congolese are concentrated in the Makindye district of Kampala (Uganda’s capital) and the Nakivale Refugee Settlement. Although a number of studies in recent years have focused on the sexual and reproductive health of refugees living in Uganda,^29–32^ little work has explored displaced populations’ abortion experiences. Our study aimed to fill this gap.

## Methods

In the summer of 2017, we undertook a multi-methods reproductive health needs assessment with Congolese refugees living in Kampala and the Nakivale Refugee Settlement.^33^ Modelled after other needs assessments conducted in refugee and displacement settings,^12–14^ our study consisted of four components: (1) a review of the published literature as well as internal reports, statistics, and documents from institutions working with refugees in Uganda; (2) interviews with well-positioned key informants; (3) focus group discussions (FGDs) with Congolese women; and (4) in-depth interviews with Congolese refugee women of reproductive age.

Interviews with key informants aimed to explore a range of perspectives from individuals and agency representatives working with refugees and/or in the SRH field. Our semi-structured interviews focused on the availability of, accessibility of, and avenues for improving reproductive health services to Congolese refugees in particular. Our key informants included policy makers, health service providers, and non-governmental organisation (NGO) representatives. We purposively recruited interviews by utilising publicly available information, study team contacts, and early participant referrals.

For both our FGDs and our in-depth interviews, we recruited Congolese women of reproductive age (15 to 49 inclusive) who resided either in Kampala or the Nakivale Refugee Settlement. We worked with two refugee-focused organisations to recruit these women and supplemented this strategy with flyers, word-of-mouth campaigns, and early participant referral. Focus group discussions with women focused on maternal health and delivery care, contraception, and abortion/post-abortion care, and explored community knowledge, access to and utilisation of SRH services, facilitators and barriers to access, and priorities for improvement. In the FGDs we aimed to solicit community norms as well as identify outliers. In-person interviews with women focused on individual-level experiences with SRH services, including abortion and post-abortion care, in both the pre-displacement and displacement periods. We also asked women to reflect on the ways in which services could be improved.

RN, a tri-lingual Congolese-Canadian master’s student at the University of Ottawa (Canada) led all components of data collection after being trained by her thesis supervisor, AMF, a medical anthropologist and medical doctor with SRH expertise. RN conducted all of the interviews with key informants, which lasted 60–90 minutes, in English and later transcribed the interviews herself. AB, a Ugandan university student, acted as a local research assistant and helped coordinate these interviews. Focus group discussions lasted an average of one hour and were conducted in French, Lingala, or Swahili; RN led the discussions with the help of a local research assistant who was able to interpret from Swahili to English. These discussions were translated into English by translators hired from the two refugee-focused organisations and transcribed verbatim. RN also led all of the in-depth interviews, which she conducted in French, Lingala, and Swahili (with assistance). These interviews lasted 30–60 minutes and were later translated and transcribed by local research assistants. We offered both the FGD and in-depth interview participants a small honorarium to reimburse them for their transportation costs and cover any childcare-related expenses.

RN took extensive notes during each interaction, debriefed with local research assistants immediately after each FGD or interview, and debriefed with AMF regularly. RN also formally memoed after each interaction, a process that allowed for reflections on emergent themes and concepts as well as the participant-researcher-interpreter interaction. The memoing process also allowed RN to establish thematic saturation for the in-depth interviews^34^; once we suspected we had reached thematic saturation, we did several additional interviews for confirmation and then closed this portion of the study.

With the permission of the participants, we audio-recorded all but one of the interviews and all discussions. We used *NVivo 11.4.3* to manage our data, which included transcripts, notes, and memos and we analysed these data for content and themes.^34–35^ We employed an iterative analytic approach and began data analysis during the data collection phase. We developed an initial codebook containing *a priori* codes based on the study aims and research questions; as we familiarised ourselves with the data, we added emergent codes and categories. RN coded the data and then worked to identify themes; AMF reviewed the codebook and a subset of transcripts and provided input on emergent codes and categories. We initially worked with each component of the study separately; in the final analytic phase we reviewed all components and explored areas of agreement and disagreement. Regular meetings between RN and AMF guided this process and the overall interpretation of the findings. Presentation of these results at several international meetings and global webinars yielded valuable feedback that shaped our final recommendations.

This project received ethics approval from the Social Sciences and Humanities Research Ethics Board at the University of Ottawa, Canada (File #: 04-17-15), the School of Medicine Research Ethics Committee at Makerere University, Uganda (File #: 2017-073), and the Uganda National Council of Science and Technology (File #: SS-4321). Additionally, given the nature of this project and the participants, we also obtained clearance from the Office of the Prime Minister in Uganda to conduct our study at the Nakivale Refugee Settlement.

In this paper, we focus specifically on the findings related to abortion and post-abortion care. We use illustrative quotes to showcase themes and ideas. To provide thick description and a more robust picture of women’s experiences with abortion and post-abortion care, we also present several narrative vignettes. These vignettes summarise the experiences of individual women who shared their stories with us, based on our close review of in-depth interview transcripts. We have removed and/or masked all personally identifying information and used pseudonyms throughout.

## Results

### Participant characteristics

The 11 key informants we spoke with included policy makers, health service providers, and NGO representatives working in the humanitarian sector or with refugees in the Nakivale Refugee Settlement (*n* = 4) and in Kampala (*n* = 7). A total of 36 married and unmarried Congolese women participated in our four FGDs. Focus group discussion participants ranged from 15 to 48 years old and were divided by marital status across both study locations as we have detailed elsewhere.^33^ We conducted 21 in-depth interviews with Congolese women in the Nakivale Refugee Settlement (*n* = 10) and Kampala (*n* = 11). Of our Nakivale Refugee Settlement participants, 40% were between the ages of 45 and 49, 50% were widowed, 40% had some high school education, and 40% originated from North Kivu. The majority of our Kampala participants were between the ages of 26 and 35, over half (56%) were married, 36% were high school graduates, and 36% were from South Kivu.

### Congolese refugees in Uganda are engaging in unsafe and ineffective abortion practices

“Here you will find a young girl of 15 years is pregnant and then because she fears giving birth, she ends up doing an abortion.” *(Unmarried FGD Participant, Nakivale Refugee Settlement)*

**Figure 1.**
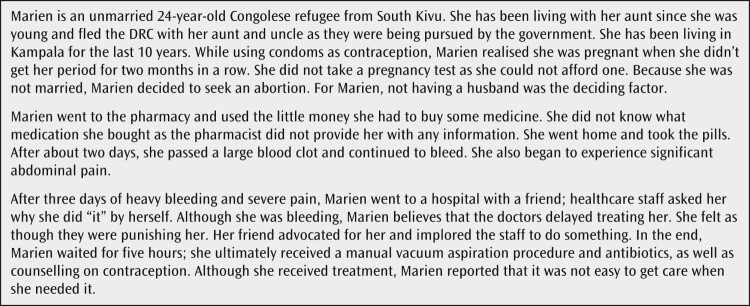
Marien’s story

Unlike Marien (Figure 1), most of our FGD and interview participants did not share personal abortion stories. However, almost all the refugee women in our study discussed the abortion experiences of women in their communities. Women described a range of practices including using detergents, crushed bottles, herbs and teas, pain medications such as paracetamol, and large doses of oral contraceptive pills

Consistent with the reports of Congolese women, key informant health service providers repeatedly discussed seeing unsafe abortion and treating abortion-related complications. As one health service professional working in the Nakivale Refugee Settlement explained: “*You cannot go a month without receiving an [unsafe] abortion case*”*.* Key informants reported that refugee women use a range of objects to instrument the uterus (including bottles and sharp sticks), detergents and vaginal douches, and non-abortifacient medications (such as antimalarials and pain medications), to induce an abortion. They were clear that the abortion practices of refugee women were similar to those of host country nationals. As one key informant from the Nakivale Refugee Settlement explained: “*[Abortions] happen, we treat them. But I can say the prevalence, or the abortion ratio [for Congolese refugees] is not far from the national*”.

### The legal restrictions on abortion directly contribute to unsafe practices

“The [legal] environment is not that friendly for people to go and seek a safe abortion. Even the service providers themselves are hesitant to provide safe abortions since they can be persecuted.” *(Key informant, Kampala)*

Key informants in our study recognised that the legal restrictions on abortion and the lack of clarity regarding the interpretation of the exceptions contribute to the occurrence of unsafe abortion. Almost all the key informants reported that, due to the laws surrounding abortion, women fear the legal consequences of attempting to have an abortion at a healthcare facility. At the same time, health service providers fear that there will be professional repercussions if they provide induced abortion care to a patient. As one key informant from Kampala stated:

“Abortion - it is actually a big problem because it’s illegal. First of all, [safe and legal abortion] is not that available. And people who provide it, it’s expensive. You find that it’s rare that you can do it in a recognised health center or government health center. So, you have to go to a private [clandestine] clinic, because no one wants to be known as [having performed an] abortion.”

### The legal restrictions on induced abortion impact post-abortion care

“[When] we have cases of abortion [most of the women] will not really come out clearly, in case it’s induced. Nobody will come out and say, ‘Ah, this is induced’. They will say, ‘Oh I got a fever and then all of a sudden, I started seeing bleeding’.” *(Key informant, Nakivale Refugee Settlement)*

**Figure 2.**
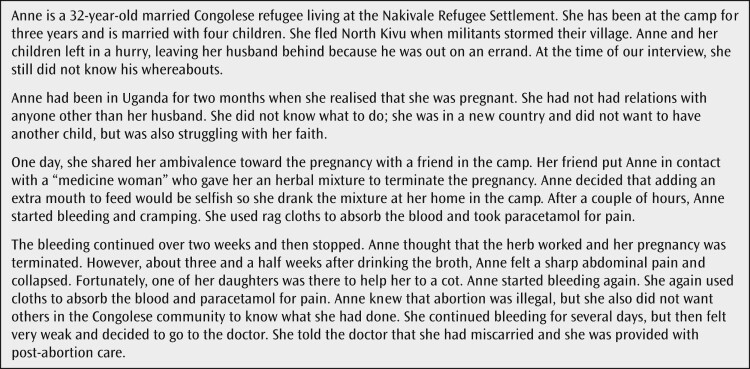
Anne’s story

The legal restrictions on induced abortion appear to be having a chilling effect on post-abortion care. Consistent with global standards, refugee women in Uganda are entitled to receive post-abortion care, as Anne's story shows (Figure 2). As one key informant in Kampala explained, “*We have the [post-abortion care] services. We have skilled personnel who provide those services to [refugees]*.”

However, the fears that both women and providers have about the legality of induced abortion appear to influence post-abortion care. Participants in the interviews and FGDs repeatedly explained that Congolese women will not present to a health facility after attempting to induce an abortion because of concerns about prosecution and imprisonment. Indeed, we heard several stories about women who postponed seeking post-abortion care until it was too late. This was echoed by a key informant from Kampala who shared the following story:

“There are those [refugee women] who induce abortions, and yet they are not well treated after. And this has so many consequences for them. I know one lady who did it [an unsafe abortion], and she did not get adequate medical treatment. Unfortunately, challenges in obtaining post-abortion care result in dire consequences such as infection and even death.”

Several of our key informants also reported being unclear as to the legal status of post-abortion care and were unsure what could be provided if a woman presented with complications from a pregnancy loss.

## Discussion

The relationship between the legal status of abortion and the safety of abortion provision is clear: restrictive abortion laws directly impact access to safe abortion and contribute to the prevalence of unsafe or clandestine abortion.^36–37^ Indeed, highly restrictive abortion laws do not reduce abortion rates – prohibitive laws only serve to increase the likelihood of unsafe abortions.^5^ The complications associated with unsafe abortion include gynecological injury, haemorrhage, infections, uterine scarring, and death.^38^

Unsafe abortion contributes to a large proportion of global maternal mortality and morbidity.^10^ Despite the global acknowledgement of the impact of unsafe abortion on maternal health outcomes, there are still many countries, largely concentrated in the Global South, with highly restrictive abortion laws.^5^ Data suggest that 97% of unsafe abortions occur in low-income countries in Africa, Asia, and Latin America.^38^ Coincidentally, developing countries, characterised by weak health systems and poverty, host the overwhelming majority of displaced populations.^1^ Although reliable data on abortion in refugee, crisis, and conflict settings is limited, humanitarian stakeholders and agencies recognise the value of abortion as a life-saving procedure^10^ and unintended pregnancy is a significant public health issue in humanitarian settings.^9,39–40^ Therefore, efforts to ensure access to safe abortion care for all women, including refugees and displaced populations, must continue.^41^ Indeed, the *2018 Interagency Field Manual for Reproductive Health in Humanitarian Settings* now provides greater clarity on the need to provide safe abortion care, to the full extent of the law, in different phases of humanitarian emergencies.^39–40^ This is an important step in increasing access to safe abortion care.

However, the legal restrictions surrounding abortion in Uganda continue to present challenges to Ugandan and refugee women alike. Unintended pregnancy is quite prevalent in Uganda^42^ and unsafe abortion remains a major problem for Ugandan women despite the decline in the abortion rate, from 54 abortions per 1000 women of reproductive age in 2003 to 39 in 2013.^24^ Our study suggests that the laws in Uganda affect the provision of safe abortion care as both women and providers fear the consequences of obtaining and providing an abortion.^19^ Reasons for their apprehension include the lack of clarity on legal exceptions and inconsistent interpretation of the criminal code, laws, and policies; these dynamics create confusion for both women and providers.^21^ As is consistent in other humanitarian settings,^12,14^ our study reveals that safe abortion care is not routinely provided in camp and urban settings.

Moreover, our study suggests that Congolese refugees engage in unsafe abortion practices, a dynamic that is consistent with other areas where safe abortion care is not available.^11^ As in other humanitarian settings,^14–16^ establishing safe referral systems that link eligible refugee women to Ugandan health facilities could expand access to safe and legal abortion care.

Our study also suggests that there is a need to engage in efforts to reduce harm from unsafe abortion. Recent projects in both northern Thailand and Nepal suggest that community-based distribution of misoprostol can be an effective strategy in low-resource and conflict-affected settings.^43–44^ Implementing a similar strategy could prove feasible in Uganda as misoprostol is currently registered to treat post-partum haemorrhage and is on the essential medicines list.^45^ Further, some civil society organisations in Uganda already encourage the off-label use of misoprostol to induce labour or abortion.^46^ In camp-based based settings, misoprostol is made available to providers through the Inter-agency Reproductive Health Kits.^39^ Thus drugs that could significantly reduce harm from unsafe abortion are available in both camp and non-camp settings.

Although abortion is legally restricted in Uganda, post-abortion care is not; a range of health facilities can treat and manage post-abortion complications.^45^ Findings from our study show that the legal status of abortion in Uganda not only affects access to induced abortion services but also access to post-abortion care, which is consistent with previous research.^45^ In consultation with experts and consistent with global standards of care,^47^ the Ministry of Health in Uganda has adopted guidelines for comprehensive abortion care that aim to address and respond to unsafe abortion through the improvement of services related to unintended and unwanted pregnancy.^48^ Thus, ensuring women are fully aware that post-abortion care is legally permissible, as well as when are how to access services, is crucial for reducing poor reproductive health outcome.

## Limitations

As is true of qualitative studies, the findings presented in this article are not representative or generalisable.^49^ Nonetheless, we are confident that the themes we have identified are transferable to other Congolese refugee populations in Uganda. It is worth noting that we recruited participants only in Kampala and at the Nakivale Refugee Settlement through NGOs; our data is not reflective of other perspectives. Finally, this study was a cross-language qualitative study which included focus group discussions and in-depth interviews with refugee women in French, Lingala and/or Swahili. Consequently, the use of interpreters may have impacted our findings as complex concepts, phrases, and words may be difficult and or impossible to translate.

We also recognise that RN’s positionality as a young Congolese-Canadian woman with formal graduate-level education influenced interactions with both refugee participants and Ugandan stakeholders. We tried to minimise this limitation by working with local NGOs and research assistants from the community. Through memoing, debriefings, and team meetings with local research assistants, we were better able to understand these influences and how to navigate these complex dynamics, thereby increasing the credibility and trustworthiness of this study.

## Conclusion

Our study shows that legal restrictions, inconsistent interpretation of conflicting and somewhat ambiguous laws and policies, and fear of legal consequences influence access to safe abortion and post-abortion care for Congolese refugees living in Uganda. While clarification of the current legal status of abortion is required, strategies to overcome legal and policy barriers are also warranted. Harm reduction efforts that increase access to safe abortion care and campaigns to increase awareness of post-abortion care services to refugee and Ugandan women could save lives and improve reproductive health outcomes.
